# A systematic review and meta-analysis of sex differences in clinical outcomes of hypertrophic cardiomyopathy

**DOI:** 10.3389/fcvm.2023.1252266

**Published:** 2023-12-05

**Authors:** Guyue Liu, Li Su, Mingjian Lang

**Affiliations:** Department of Cardiology, Chengdu Fifth People’s Hospital, Chengdu, China

**Keywords:** hypertrophic cardiomyopathy, meta-analysis, clinical outcome, sex differences, female, male

## Abstract

**Background:**

Hypertrophic cardiomyopathy (HCM) is recognized as the most prevalent form of genetic cardiomyopathy, and recent investigations have shed light on the existence of sex disparities in terms of clinical presentation, disease progression, and outcomes.

**Objectives:**

This study aimed to systematically review the literature and perform a meta-analysis to comprehensively compare the clinical outcomes between female and male patients with HCM.

**Methods:**

A thorough search was conducted in databases including PubMed, Embase, Cochrane Library, and Web of Science, encompassing literature from inception until June 2023. The primary endpoints examined were: (1) all-cause mortality; (2) an arrhythmic endpoint comprising sudden cardiac death (SCD), sustained ventricular tachycardia, ventricular fibrillation, or aborted SCD; and (3) a composite endpoint incorporating either (1) or (2), in addition to hospitalization for heart failure or cardiac transplantation. Pooled estimates were derived using a random-effects meta-analysis model.

**Results:**

The analysis encompassed a total of 29 observational studies, involving 44,677 patients diagnosed with HCM, of which 16,807 were female. Baseline characteristics revealed that the female group exhibited an advanced age [55.66 ± 0.04 years vs. 50.38 ± 0.03 years, pooled mean difference (MD) = 0.31, 95% CI: 0.22–0.40, *p* = 0.000, *I^2^* = 88.89%], a higher proportion of New York Heart Association class III/IV patients [pooled odds ratio (OR) = 1.94, 95% CI: 1.55–2.43, *p* = 0.000, *I^2^* = 85.92%], and a greater prevalence of left ventricular outflow tract gradient greater than or equal to 30 mmHg (pooled OR = 1.48, 95% CI: 1.27–1.73, *p* = 0.000, *I^2^* = 68.88%) compared to the male group. The female group were more likely to have a positive genetic test (pooled OR = 1.27, 95% CI: 1.08–1.48, *p* = 0.000, *I^2^* = 42.74%) and to carry the myosin heavy chain beta 7 mutation (pooled OR = 1.26, 95% CI: 1.04–1.54, *p* = 0.020, *I^2^* = 0.00%) compared to the male group. Female sex exhibited a significant association with increased risks of all-cause mortality (pooled OR = 1.62, 95% CI: 1.38–1.89, *p* = 0.000, *I^2^* = 72.78%) and the composite endpoint (pooled OR = 1.47, 95% CI: 1.20–1.79, *p* = 0.000, *I^2^* = 84.96%), while no substantial difference was observed in the arrhythmic endpoint (pooled OR = 1.08, 95% CI: 0.87–1.34, *p* = 0.490, *I^2^* = 55.48%).

**Conclusions:**

The present findings suggest that female patients with HCM tend to experience poorer clinical outcomes. It is imperative to critically reevaluate disease definitions and enhance awareness to mitigate delays in the diagnosis and treatment of HCM in women, thereby fostering equitable healthcare practices.

**Systematic Review Registration:**

https://www.crd.york.ac.uk/, PROSPERO (CRD42023431881).

## GRAPHICAL ABSTRACT

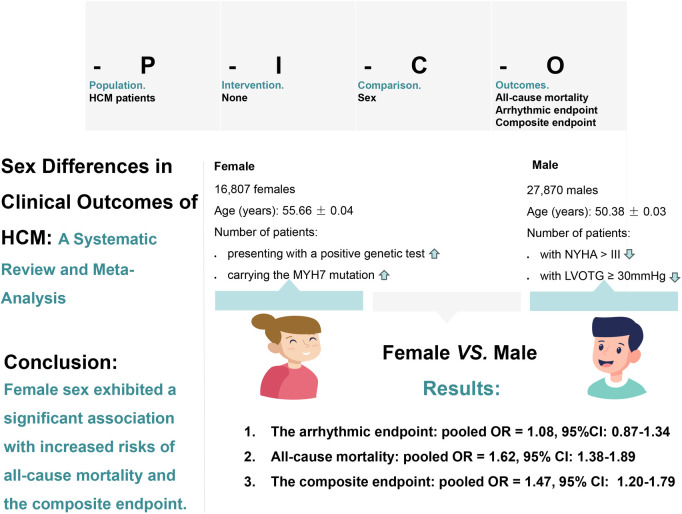

## 1. Introduction

Hypertrophic cardiomyopathy (HCM) is primarily caused by genetic variants affecting sarcomere proteins and inherited in an autosomal dominant manner (1). As a result, the prevalence of HCM in the general population is expected to be similar between the sexes (1). Nevertheless, recent investigations have revealed notable sex disparities in the clinical manifestation, progression, and prognosis of HCM. Specifically, females tend to receive a diagnosis at an older age (≥65 years) and exhibit a higher burden of symptoms (2). Furthermore, they demonstrate a greater prevalence of family history associated with HCM (2). Additionally, females present a heightened frequency of left ventricular outflow obstruction (2). In terms of treatment, women with hypertrophic obstructive cardiomyopathy (HOCM) are less likely to be prescribed beta blockers, angiotensin-converting enzyme inhibitors, or anticoagulants, and have lower rates of implantable cardioverter-defibrillator (ICD) utilization (3). Furthermore, women face an increased risk of heart failure and mortality (3). While Josef Veselka et al. reported a comparable 10-year freedom from all-cause mortality between women and men (76% vs. 80%) following alcohol septal ablation in HOCM (4). Given the divergent results among these studies, this systematic review and meta-analysis was conducted to comprehensively compare the clinical outcomes between females and males in patients diagnosed with HCM.

## 2. Methods

### 2.1. Search strategy

A comprehensive systematic literature review was performed in June 2023, utilizing prominent databases including PubMed, Embase, Cochrane Library, and Web of Science. The search strategy employed a combination of relevant medical subject headings and keywords to identify pertinent articles. PubMed search was conducted with terms like “Cardiomyopathy, Hypertrophic,” “Cardiomyopathies, Hypertrophic,” “Hypertrophic Cardiomyopathies,” “Hypertrophic Cardiomyopathy,” “Cardiomyopathy, Hypertrophic Obstructive,” “Cardiomyopathies, Hypertrophic Obstructive,” “Hypertrophic Obstructive Cardiomyopathies,” “Hypertrophic Obstructive Cardiomyopathy,” “Obstructive Cardiomyopathies, Hypertrophic,” “Obstructive Cardiomyopathy, Hypertrophic,” “Familial hypertrophic cardiomyopathy,” “HCM,” or “HOCM,” in association with “Sex,” “Gender,” “Woman,” “Women,” “Man,” “Men,” “Female,” “Male,” “Females,” or “Males.” The same search terms were suitably adapted for each respective database (see Supplementary Table S1 for detailed information). Furthermore, an additional manual search of the reference lists of selected studies and relevant meta-analyses was conducted to identify any potentially eligible studies that may have been missed. To ensure a rigorous and unbiased selection process, two authors (G. L. and L. S.) independently evaluated the retrieved articles for inclusion. In cases where discrepancies arose, a consensus was reached through discussion involving all authors.

### 2.2. Inclusion criteria

The eligibility criteria encompassed the following aspects: (1) Only studies adhering to well-established research designs were considered for inclusion, including cohort studies (prospective or retrospective), case-control studies, experimental studies, or randomized controlled trials. Specifically, the focus of these studies was on hypertrophic cardiomyopathy (HCM) patients, with a primary objective of comparing clinical outcomes between females and males. To maintain methodological robustness, case series, case reports, dissertations, and conference proceedings were excluded from consideration within this review. (2) Syndromic forms of HCM and systemic diseases capable of producing the observed magnitude of hypertrophy (such as aortic stenosis, uncontrolled arterial hypertension) were excluded from the analysis. (3) There were no restrictions on sample size. (4) Only full articles written in English were taken into account.

### 2.3. Data extraction and quality assessment tool

Data extraction from the included studies was performed independently by two authors (G.L. and L.S.). In case of any discrepancies, a consensus was reached through discussion involving a third author (M.L.). The extraction process involved obtaining relevant information pertaining to both continuous and dichotomous variables from each cohort. For continuous variables, including means or medians, the corresponding values were extracted. Similarly, for dichotomous variables, absolute numbers or percentages were recorded for each cohort. In instances where the studies did not directly provide continuous or dichotomous variables, effect sizes were extracted.

The quality assessment of the included observational studies was conducted using the Newcastle-Ottawa Scale (NOS), a widely accepted tool for appraising the methodological quality of non-randomized studies. The NOS employs a star system ranging from 0 to 9, with higher scores indicating superior study quality. Each study was rigorously evaluated across three key domains: the selection of study groups, the comparability of these groups, and the ascertainment of outcomes.

### 2.4. Baseline characteristics and outcome measures

Baseline characteristics of interest in the patient cohort encompassed several key factors, including age, history of syncope, history of hypertension, family history of sudden cardiac death (SCD), family history of HCM, invasive procedures during the course of HCM (septal reduction therapy or use of ICDs), New York Heart Association (NYHA) class III/IV, presence of non-sustained ventricular tachycardia (NSVT), atrial fibrillation (AF), the number of patients with left ventricular outflow tract gradient (LVOTG) greater than or equal to 30 mmHg, maximal wall thickness (MWT), left ventricular ejection fraction (LVEF),left atrial diameter (LAD), the number of patients with positive genetic tests (genes involved include myosin-binding protein C (MYBPC3), myosin heavy chain beta 7 (MYH7), essential and regulatory myosin light chains (MYL2, MYL3), cardiac troponin T (TNNT2), cardiac troponin I (TNNI3), a-tropomyosin (TPM1) and cardiac actin (ACTC)), number of patients carrying the MYBPC3 and MYH7 mutations.

The prespecified study endpoints consisted of three primary outcomes: (1) all-cause mortality; (2) an arrhythmic endpoint encompassing SCD, sustained ventricular tachycardia (SVT), sustained ventricular fibrillation (SVF), or aborted SCD, which included instances of successful resuscitation following cardiac arrest and appropriate ICD shocks, and (3) a composite endpoint comprising either endpoint (1) or (2), along with hospitalization for heart failure (HF) or cardiac transplantation. SCD was strictly defined as an unexpected and instantaneous fatality, while aborted SCD referred to cases in which successful resuscitation occurred following cardiac arrest.

To address the existing heterogeneity across the included studies, a predefined meta-regression analysis and subgroup analysis were conducted. These analyses focused on crucial variables, including mean age (<50 years old or ≥50 years old), follow-up time (<3 years or ≥3 years), sample size (<1,000 or ≥1,000), postoperative patients (yes or no), data matching (yes or no), and the type of estimated effect size (odds ratio, relative risk, or hazard ratio).

### 2.5. Meta-analysis

The study design and findings were reported in accordance with the guidelines outlined in the Preferred Reporting Items for Systematic Review and Meta-Analysis (PRISMA) 2020 checklist, ensuring the comprehensive and transparent reporting of the research process (see Supplementary Table S2 for detailed information).

Meta-analysis was conducted using Stata version 17.0 (StataCorp, College Station, Texas). Random-effects models were employed to estimate pooled effect sizes, and the resulting outcomes were presented as odds ratios (ORs) and mean differences (MDs), providing a robust measure of the collective findings.

To assess the presence of heterogeneity among the included studies, statistical measures including the *I*-squared (*I^2^*) statistic and Cochrane Q-statistic were utilized. Heterogeneity was considered significant if the *I^2^* value exceeded 50% or if the *p*-value associated with the Q-statistic was less than 0.1. In order to explore potential sources of heterogeneity, both meta-regression analysis and subgroup analysis were performed, considering relevant variables that could contribute to the observed heterogeneity.

The robustness of the results was evaluated through sensitivity analysis, whereby each study was sequentially excluded to assess its influence on the overall outcomes. A consistent pattern of results would indicate the reliability of the findings.

Statistical significance was defined as a *p*-value less than 0.05, indicating a meaningful effect or association. These rigorous analytical approaches and assessments were implemented to ensure the validity, reliability, and robustness of the study's findings, thereby enhancing the credibility of the research outcomes.

## 3. Results

### 3.1. Literature search

**Figure 1:**
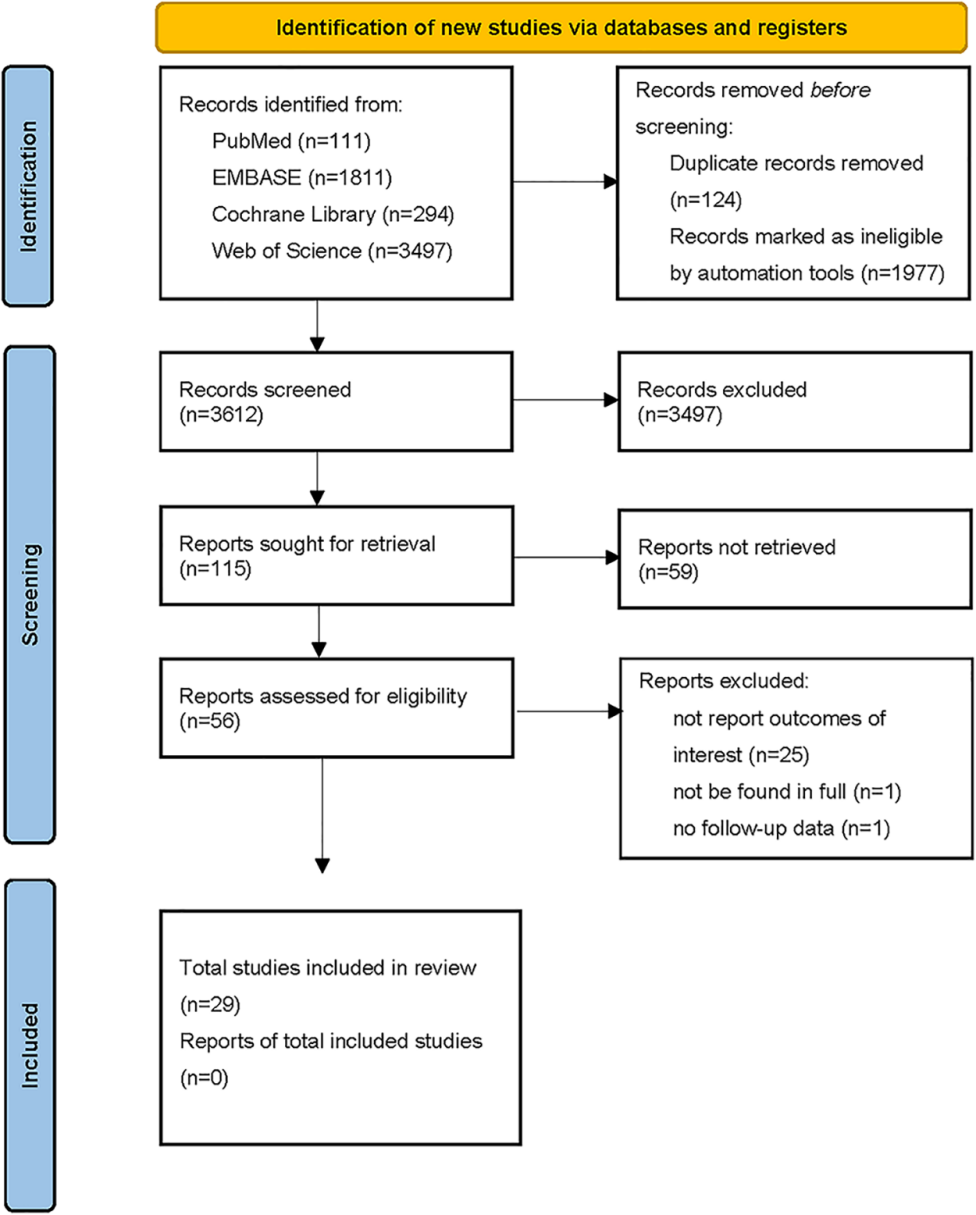
Flow chart of studies selection process.

The flowchart depicting the selection process of the meta-analysis is presented in Figure 1. A systematic search of relevant databases yielded an initial total of 5,713 records. After removing duplicates, non-English articles, and studies that did not meet the predetermined inclusion and exclusion criteria (e.g., reviews, case reports, editorials, opinion letters, dissertations, conference proceedings, and original articles that did not align with the research objectives), 56 articles were subjected to a thorough evaluation. Following this screening process, one article could not be accessed in its entirety, one article lacked the necessary follow-up data, and twenty-five articles did not report the specific outcomes of interest. Ultimately, a total of twenty-nine retrospective cohort studies were considered eligible for quantitative analysis (5–33).

### 3.2. Study characteristics and quality assessment tool

Table 1 presents a comprehensive overview of the included studies, providing valuable insights into potential biases that may influence our interpretations. In this meta-analysis, we included a total of 29 observational studies conducted between 2004 and 2022. The analysis encompassed a large sample size of 44,677 patients diagnosed with HCM, with 16,807 females and 27,870 males. The studies varied in terms of participant enrollment, ranging from the largest study with 5,873 participants to the smallest study with 50 participants. The mean age of the patients ranged from 44.9 to 63.0 years, and the follow-up duration ranged from 30 days to 18.1 years.

**Table 1:** Baseline characteristics of the included studies.

First author	Country/study period	Inclusion criteria^a^	Female/male		Matching	Characteristic differences between study groups	Follow-up time, years	Outcomes reported: (1), (2), (3)
			*n*	Age, years Mean (SD)/IQR				
Ghiselli et al. (5)	Italy 2004–2016	Yes	65/177	51 (16)/44 (17)	–	Higher age, less HOCM in women	5.9 ± 4.2	(1), (2), (3)
Ho et al. (6)	China 1973–2001	Yes	56/62	56 (14)/52 (13)	–	Two well matched cohorts	5.8 ± 4.3	(1)
Huang et al. (7)	China 2008–2016	Yes	260/316	57.2 (16.7)/53.0 (15.7)	–	Higher age, NYHA class, and LVOTG at rest, more moderate-severe MR in women	3.2 ± 2.3	(1), (3)
Huurman et al. (8)	Netherlands 2000–2019	Yes	63/99	57 (15)/49 (14)	–	Higher age and LVOTG at rest or provocation in women	5.9 (IQR 3.0–9.1)	(1), (2), (3)
Javidgonbadi et al. (9)	Sweden 2002–2013	Yes	83/83	56 (26)/56 (25)	Age	Higher NYHA class and indexed IVS in women	18.1 ± 9.4	(1), (2)
Kim et al. (10)	Korea 2010–2016	Not mentioned	1,269/4,299	51.2 (10.4)/50.4 (9.5)	–	Higher age in women	4.4 (IQR 2.2–9.9)	(1), (3)
Lakdawala et al. (11)	USA	Yes	2,226/3,647	49.0 (IQR 36.8–65.3)/42.9 (IQR32.4–58.6)	–	Higher age, NYHA class, indexed wall thickness, EF, and LVOTG at rest in women	7.7 (IQR 3.1–15.4)	(1), (2)
Lawin et al. (12)	Germany 2002–2020	Yes	645/722	66.0 (IQR55.0–74.0)/54.0 (IQR45.0–62.0)	–	Higher age and indexed IVS diameter in women	6 months	(1), (2)
Lee et al. (13)	Korea 2007–2019	Yes	223/612	59.9 ± 13.5/54.9 ± 11.4	–	Higher age and indexed LV wall thickness in women	6.4 (IQR4.1–9.2)	(1), (2), (3)
Lu et al. (14)	USA 2015–2016	Yes	200/200	55 ± 14/55 ± 14	Age	Higher EF and LVOTG at rest in women	2.1 (IQR0.9–4.9)	(1), (2), (3)
Meghji et al. (15)	USA 1961–2016	Not mentioned	1,127/1,379	59.5 (IQR46.6–68.2)/52.9 (42.9–62.7)	–	Higher age, NYHA class, LVOTG at rest or provocation and EF, lower indexed LV mass, more moderate or severe MR in women	8.2 (IQR3.1–13.2)	(1), (2)
Montenegro et al. (16)	Portugal 1975–2015	Yes	429/613	56 ± 16/51 ± 15	–	Higher age and heart failure, more moderate or severe MR in women	65 ± 75 months	(1), (2)
Olivotto et al. (17)	Italy and USA	HCM, LV wall thickness ≥13 mm; HOCM, resting LVOTG ≥30 mmHg	393/576	51 ± 22/42 ± 18	–	Higher age, NYHA class, and LVOTG, lower LV wall thickness in women	6.2 ± 6.1	(1), (2)
Osman et al. (18)	USA 2015–2018	Not mentioned	1,170/1,127	61 (IQR52–70)/60 (IQR51–68)	Propensity score-matched	Two well matched cohorts	30 days	(1)
Rowin et al. (19)	USA 2001–2016	HCM, LV wall thickness >13 mm	794/1,329	50 ± 19/44 ± 16	–	Higher age, NYHA class, and EF, lower LV wall thickness, and LVOTG at rest or provocation in women	3.9 (IQR2.0–6.9)	(1), (2)
Terauchi et al. (20)	Japan	Yes	23/27	50 ± 19/45 ± 14	–	Higher NYHA class in women	13 ± 8	(1), (2), (3)
VanVelzen et al. (21)	Netherlands 1977–2017	Yes	387/620	56 ± 16/49 ± 15	–	Higher age and indexed LV wall thickness, more HOCM, lower LV systolic and diastolic function in women	6.8 (IQR3.2–10.9)	(1), (2), (3)
Wang et al. (22)	China 1999–2011	Yes	161/460	49.6 ± 17.2/46.714.4±	–	Higher age, more HOCM, higher NYHA class in women	4.0 (IQR2–7)	(1), (2)
Wang et al. (23)	China	Yes	162/158	50.7 ± 6.8/52.6 ± 7.3	–	Higher NYHA class in women	7.2 ± 3.9/7.9 ± 4.7	(1), (2)
Geske et al. (33)	USA1975–2012	Yes	1,661/2,012	59 ± 16/52 ± 15	–	Higher age, NYHA class, and EF, more HOCM, lower LV wall thickness in women	10.9 (IQR7.4–16.2)	(1)
Hutt et al. (24)	USA 2002–2018	Yes	995/1,124	55 ± 13	–	–	5.4 (IQR2.8–9.2)	(1), (2), (3)
Jang et al. (25)	Korea 2012–2017	Not mentioned	61/141	70 ± 12/59 ± 14	–	Higher age and NYHA class in women	34 (IQR16.1–56.8) months	(1), (3)
Lee et al. (26)	China 1990–2005	Yes	78/85	64.8 ± 11.3/57.2 ± 12.9	–	Higher age, more HOCM in women	5.4 ± 4.1	((1)
Woo et al. (27)	Canada 1978–2002	Yes	135/203	47 ± 14	–	–	7.7 ± 5.7	(1)
Ho et al. (28)	USA 1960–2016	HCM, LV wall thickness ≥13 mm	1,699/2,892	45.8(IQR30.9–58.1)	–	–	2.9 (IQR0.3–7.9)	(2)
Ball et al. (29)	Canada 1986–2007	HCM, LV wall thickness ≥13 mm	284/365	51 ± 16	–	–	7.2 ± 5.5	(1)
Lorenzini et al. (30)	United Kingdom 1980–2013	Yes	1,767/3,126	52.9 ± 17.1/47.1 ± 15.6	–	Higher age, NYHA class, and EF, more HOCM in women	6.2 (IQR3.1–9.8)	(1), (2), (3)
Kubo et al. (31)	Japan 2004–2013	Yes	196/197	63 ± 14	–	–	6.1 ± 3.2	(1), (2), (3)
Choi et al. (32)	Korea 2007–2017	Yes	179/551	57.1 ± 14.3	–	–	4,288 person-years	(2)

HOCM, obstructive hypertrophic cardiomyopathy; HCM, hypertrophic cardiomyopathy; NYHA, New York Heart Association; EF, ejection fraction; LVOTG, left ventricular outflow tract gradient; IVS, interventricular septum; MR, mitral regurgitation; HF, heart failure; AF, atrial fibrillation; SVT/SVF, sustained ventricular tachycardia/fibrillation; SCD, sudden cardiac death; SM, septal myectomy; ASA, alcohol septal ablation; LV, left ventricular; IQR, inter-quartile range; SD, standard deviation; USA, the United States of America.

^a^ Inclusion criteria: HCM, IVS ≥15 mm; HOCM, resting LVOTG ≥30 mmHg or >50 mmHg with provocation. Outcomes reported: (1) all-cause mortality; (2) an arrhythmic endpoint encompassing SCD, SVT, SVF, or aborted SCD, which included instances of successful resuscitation following cardiac arrest and appropriate implantable cardioverter-defibrillator shocks, and (3) a composite endpoint comprising either endpoint (1) or (2), along with hospitalization for heart failure or cardiac transplantation.

The NOS assessment revealed that all 29 studies were of good quality, with no studies classified as poor quality (see Supplementary Table S3 for detailed information).

### 3.3. Meta-analysis results

#### 3.3.1. Comparison estimates of baseline characteristics

Table 2 presents a comprehensive depiction of the baseline characteristics of each study, while Table 3 provides the estimated effect sizes between the female and male groups. The female group exhibited a higher mean age [55.66 ± 0.04 years vs. 50.38 ± 0.03 years, pooled mean difference (MD) = 0.31, 95% CI: 0.22–0.40, *p* = 0.000, *I^2^*^ ^= 88.89%], a higher proportion of New York Heart Association class III/IV patients [pooled odds ratio (OR) = 1.94, 95% CI: 1.55–2.43, *p* = 0.000, *I^2^*^ ^= 85.92%] and a higher prevalence of LVOTG greater than or equal to 30 mmHg (pooled OR = 1.48, 95% CI: 1.27–1.73, *p* = 0.000, *I^2^*^ ^= 68.88%), compared to the male group. The female group were more likely to have a positive genetic test (pooled OR = 1.27, 95% CI: 1.08–1.48, *p* = 0.000, *I^2^* = 42.74%) and to carry the MYH7 (pooled OR = 1.26, 95% CI: 1.04–1.54, *p* = 0.020, *I^2^* = 0.00%) than male group, but there was no significant difference between the two groups for the MYBPC3 (pooled OR = 0.99, 95% CI: 0.58–1.71, *p* = 0.980, *I^2^* = 72.96%). In terms of LVEF, the female group demonstrated a slightly higher value compared to the male group (66.69 ± 0.03% vs. 65.38 ± 0.02%), with a pooled MD of 0.08 (95% CI: 0.01–0.15, *I^2^*^ ^= 77.15%) than the male group, albeit with a *p*-value approaching significance (*p* = 0.036). Conversely, the female group exhibited lower MWT (18.49 ± 0.01 mm vs. 19.11 ± 0.01 mm, pooled MD = −0.11, 95% CI: −0.15 to −0.08, *p* = 0.000, *I^2 ^*= 13.6%) and lower LAD (41.81 ± 0.02 mm vs. 43.54 ± 0.01 mm, pooled MD = −0.14, 95% CI: −0.23 to −0.06, *p* = 0.000, *I^2 ^*= 79.39%), compared to the male group. More women with history of hypertension (pooled OR = 1.27, 95% CI: 1.19–1.37, *p* = 0.000, *I^2^*^ ^= 21.22%) and septal reduction therapy (pooled OR = 1.52, 95% CI: 1.27–1.81, *p* = 0.000, *I^2^*^ ^= 47.80%). However, no statistically significant differences were observed between the female and male groups in terms of history of syncope (pooled OR = 1.08, 95% CI: 0.94–1.25, *p* = 0.290, *I^2 ^*= 59.06%), NSVT (pooled OR = 1.04, 95% CI: 0.70–1.54, *p* = 0.845, *I^2 ^*= 91.54%), use of ICDs (pooled OR = 0.93, 95% CI: 0.76–1.14, *p* = 0.490, *I*^2^ = 61.48%), and AF (pooled OR = 1.14, 95% CI: 0.99–1.32, *p* = 0.080, *I^2 ^*= 54.37%). There was also no difference between the two groups in terms of family history of SCD (pooled OR = 1.00, 95% CI: 0.72–1.40, *p* = 0.975, *I^2 ^*= 92.91%) or family history of HCM (pooled OR = 0.99, 95% CI: 0.76–1.28, *p* = 0.908, *I^2 ^*= 88.35%).

**Table 2:** Baseline patients’ characteristics of interest in each study.

First author	Age, years		Syncope, *n*		Family history of SCD, *n*		Family history of HCM, *n*		NYHA >III, *n*		NSVT, *n*		AF, *n*		LVOTG ≥30 mmHg, *n*	
	Female	Male	Female	Male	Female	Male	Female	Male	Female	Male	Female	Male	Female	Male	Female	Male
Ghiselli et al. (5)	51	44	11	31	19	37	38	77	9	13	8	22	8	23	8	74
Ho et al. (6)	56	52	3	11	**–**	**–**	7	10	14	14	**–**	**–**	**–**	**–**	12	6
Huang et al. (7)	57.2	53	73	106	**–**	**–**	**–**	**–**	122	97	**–**	**–**	48	34	140	146
Huurman et al. (8)	57	49	**–**	**–**	**–**	**–**	**–**	**–**	50	76	**–**	**–**	12	20	63	99
Javidgonbadi et al. (9)	57	56	23	18	17	9	**–**	**–**	40	29	7	8	4	4	64	64
Kim et al. (10)	51.2	50.4	**–**	**–**	**–**	**–**	**–**	**–**	**–**	**–**	**–**	**–**	38	121	**–**	**–**
Lakdawala et al. (11)	49	42.9	**–**	**–**	**–**	**–**	2,009	3,297	237	171	**–**	**–**	**–**	**–**	696	920
Lawin et al. (12)	66	54	167	130	158	179	**–**	**–**	500	428	**–**	**–**	**–**	**–**	**–**	**–**
Lee et al. (13)	59.9	54.9	36	80	36	71	25	52	44	110	18	12	29	81	43	80
Lu et al. (14)	55	55	44	39	54	50	34	31	40	15	19	28	**–**	**–**	**–**	**–**
Meghji et al. (15)	59.5	52.9	219	252	187	203	239	236	1,023	1,169	91	139	204	282	1,127	1,379
Montenegro et al. (16)	56.4	51.2	39	56	114	117	149	175	29	16	74	129	128	160	160	205
Olivotto et al. (17)	51	42	72	57	**–**	**–**	56	86	70	21	**–**	**–**	21	28	144	131
Osman et al. (18)	61	60	**–**	**–**	**–**	**–**	**–**	**–**	**–**	**–**	**–**	**–**	**–**	**–**	**–**	**–**
Rowin et al. (19)	55	49	85	174	97	129	218	292	100	220	312	306	**–**	–	357	408
Terauchi et al. (20)	50	45	3	1	**–**	**–**	**–**	**–**	4	0	**–**	**–**	**–**	**–**	5	3
VanVelzen et al. (21)	56	49	10	29	**–**	**–**	**–**	**–**	**–**	**–**	**–**	**–**	10	11	140	160
Wang et al. 2014, (22)	49.6	46.7	39	110	25	56	41	99	**–**	**–**	**–**	**–**	19	59	72	149
Wang et al. (23)	59	52.6	28	22	9	10	33	30	109	89	**–**	**–**	38	23	158	162
Geske et al. (33)	–	52	236	309	242	302	392	422	748	710	41	74	1,661	367	905	925
Hutt et al. (24)	70	–	–	–	–	–	–	–	–	–	**–**	**–**	–	–	–	–
Jang et al. (25)	64.8	59	2	6	2	5	1	2	9	3	**–**	**–**	61	23	–	–
Lee et al. (26)	–	57.2	10	11	–	–	–	–	–	–	**–**	**–**	–	–	51	28
Woo et al. (27)	–	–	–	–	–	–	–	–	–	–	**–**	**–**	–	–	–	–
Ho et al. (28)	–	–	–	–	–	–	–	–	–	–	**–**	**–**	–	–	–	–
Ball et al. (29)	52.9	–	–	–	–	–	–	–	–	–	**–**	**–**	–	–	–	–
Lorenzini et al. (30)	–	47.1	289	436	467	660	–	–	288	226	296	–	1,767	939	–	–
Kubo et al. (31)	–	–	–	–	–	–	–	–	–	–	**–**	–	–	–	–	–
Choi et al. 2019, (32)	57.1	–	–	–	–	–	–	–	–	–	**–**	–	–	–	–	–

First author	MWT, mm		LVEF, %		LAD, mm		Hypertension, *n*		Invasive procedures, *n* (Septal reduction therapy/Use of ICDs)		Genetic testing, *n*, positive/total		MYBPC3, *n*		MYH7, *n*	
	Female	Male	Female	Male	Female	Male	Female	Male	Female	Male	Female	Male	Female	Male	Female	Male
Ghiselli et al. (5)	20	21	67	67	40	43	20	46	5/-	3/-	41/64	96/160	–	–	–	–
Ho et al. (6)	18	18	68	72	38	37	0	0	–	–	–	–	–	–	–	–
Huang et al. (7)	19.2	19.9	63.9	66.4	41.5	40.5	97	96	31/-	19/-	–	–	–	–	–	–
Huurman et al. (8)	19	20	**–**	**–**	46	48	29	27	63/7	99/15	–	–	–	–	–	–
Javidgonbadi et al. (9)	18	20	70	65	39.1	43.4	–	–	-/5	-/8	–	–	–	–	–	–
Kim et al. (10)	**–**	**–**	**–**	**–**	**–**	**–**	0	0	**–**	**–**	**–**	**–**	–	–	–	–
Lakdawala et al. (11)	17.8	18.5	66	64.6	41.5	43.4	242	306	-/492	-/752	717/1,410	1,030/2,378	373	618	223	279
Lawin et al. (12)	20	21	**–**	**–**	**–**	**–**	–	–	645/58	722/84	**–**	**–**	–	–	–	–
Lee et al. (13)	17.1	17	64.7	64.4	44.7	44.3	99	252	–	–	–	–	–	–	–	–
Lu et al. (14)	20	21	67	64	40	44	104	101	69/-	51/-	33/48	51/78	8	14	5	7
Meghji et al. (15)	19	20	73	70	**–**	**–**	586	652	1,127/-	1,379/-	**–**	**–**	–	–	–	–
Montenegro et al. (16)	19.4	19.5	65.6	64.3	**–**	**–**	217	264	41/46	45/94	**–**	–	–	–	–	–
Olivotto et al. (17)	21	22	**–**	**–**	41	42	–	**–**	43/40	37/41	–	–	–	–	–	–
Osman et al. (18)	**–**	**–**	**–**	**–**	**–**	**–**	866	802	1,170/-	1,127/-	**–**	**–**	–	–	–	–
Rowin et al. (19)	18.7	19.1	64	63	40	42	268	431	372/25	405/57	74/123	101/228	37	51	24	28
Terauchi et al. (20)	20	21	67	65	40	41	–	**–**	–	–	23/23	27/27	–	–	–	–
VanVelzen et al. (21)	18	19	**–**	–	44	45	146	164	-/5	-/15	153/299	277/511	–	–	–	–
Wang et al. (22)	21	21	67.1	67.5	**–**	**–**	0	0	28/1	60/4	**–**	**–**	–	–	–	–
Wang et al. (23)	20	20	62	62	39.2	40.3	21	25	162/-	158/-	–	–	–	–	–	–
Geske et al. (33)	17.8	18.3	71	69	–	–	816	874	578/-	619/-	–	–	–	–	–	–
Hutt et al. (24)	–	–	–	–	–	–	–	–	–	–	–	–	–	–	–	–
Jang et al. (25)	19	19.2	65.1	64.9	46.9	44.8	34	70	–	–	–	–	–	–	–	–
Lee et al. (26)	18.8	18.2	–	–	38.5	37.8	14	16	–	–	–	–	–	–	–	–
Woo et al. (27)	–	–	–	–	–	–	–	–	135/-	203/-	–	–	–	–	–	–
Ho et al. (28)	–	–	–	–	–	–	–	–	–	–	504/1,008	775/1,755	–	–	–	–
Ball et al. (29)	–	–	–	–	–	–	–	–	–	–	–	–	–	–	–	–
Lorenzini et al. (30)	18	19	66	65	43	44.8	616	830	160/281	230/535	–	–	–	–	–	–
Kubo et al. (31)	–	–	–	–	–	–	–	–	–	–	–	–	–	–	–	–
Choi et al. (32)	–	–	–	–	–	–	–	–	-/8	-/8	–	–	–	–	–	–

SCD, sudden cardiac death; HCM, hypertrophic cardiomyopathy; NYHA, New York Heart Association; NSVT, non-sustained ventricular tachycardia; AF, atrial fibrillation; LVOTG, left ventricular outflow tract gradient; MWT, maximal wall thickness; LVEF, left ventricular ejection fraction; LAD, left atrial diameter; ICD, implantable cardioverter-defibrillator; MYBPC3, myosin binding protein C; MYH7, myosin heavy chain beta 7.

**Table 3:** Pooled effect estimates of baseline characteristics.

Characteristic	WMD ± SE		Estimate used	Heterogeneity estimate		Pooled estimate	95% CI	*p-v*alue
	Female	Male		*I^2^* (%)	*p*-value			
Age, years	55.66 ± 0.04	50.38 ± 0.03	MD	88.89	0.000	0.31	0.22–0.40	**0**.**000**
MWT, mm	18.49 ± 0.01	19.11 ± 0.01	MD	13.6	0.304	−0.11	−0.15 to −0.08	**0**.**000**
LVEF, %	66.69 ± 0.03	65.38 ± 0.02	MD	77.15	0.000	0.08	0.01–0.15	**0**.**036**
LAD, mm	41.81 ± 0.02	43.54 ± 0.01	MD	79.39	0.000	−0.14	−0.23 to −0.06	**0**.**000**
Syncope	–		OR	59.06	0.000	1.08	0.94–1.25	0.290
Family history of SCD	–		OR	92.91	0.000	1.00	0.72–1.40	0.975
Family history of HCM	–		OR	88.35	0.000	0.99	0.76–1.28	0.908
NYHA >III	–		OR	85.92	0.000	1.94	1.55–2.43	**0**.**000**
NSVT	–		OR	91.54	0.000	1.04	0.70–1.54	0.845
AF	–		OR	54.37	0.006	1.14	0.99–1.32	0.080
LVOTG ≥30 mmHg	–		OR	68.88	0.000	1.48	1.27–1.73	**0**.**000**
Hypertension	–		OR	21.22	0.196	1.27	1.19–1.37	**0**.**000**
Septal reduction therapy	–		OR	47.80	0.020	1.52	1.27–1.81	**0**.**000**
Use of ICDs	–		OR	61.48	0.000	0.93	0.76–1.14	0.490
Genetic testing positive	–		OR	42.74	0.106	1.27	1.08–1.48	**0**.**000**
MYBPC3	–		OR	72.96	0.025	0.99	0.58–1.71	0.980
MYH7	–		OR	0.00	0.546	1.26	1.04–1.54	**0**.**020**

WMD, weighted mean difference; SE, standard error; CI, confidence interval; MD, mean difference; OR, odds ratio; SCD, sudden cardiac death; HCM, hypertrophic cardiomyopathy; NYHA, New York Heart Association; NSVT, non-sustained ventricular tachycardia; AF, atrial fibrillation; LVOTG, left ventricular outflow tract gradient; MWT, maximal wall thickness; LVEF, left ventricular ejection fraction; LAD, left atrial diameter; ICD, implantable cardioverter-defibrillator; MYBPC3, myosin binding protein C; MYH7, myosin heavy chain beta 7.

Bold values indicate statistical significance.

#### 3.3.2. Comparison estimates of clinical outcomes

**Figure 2:**
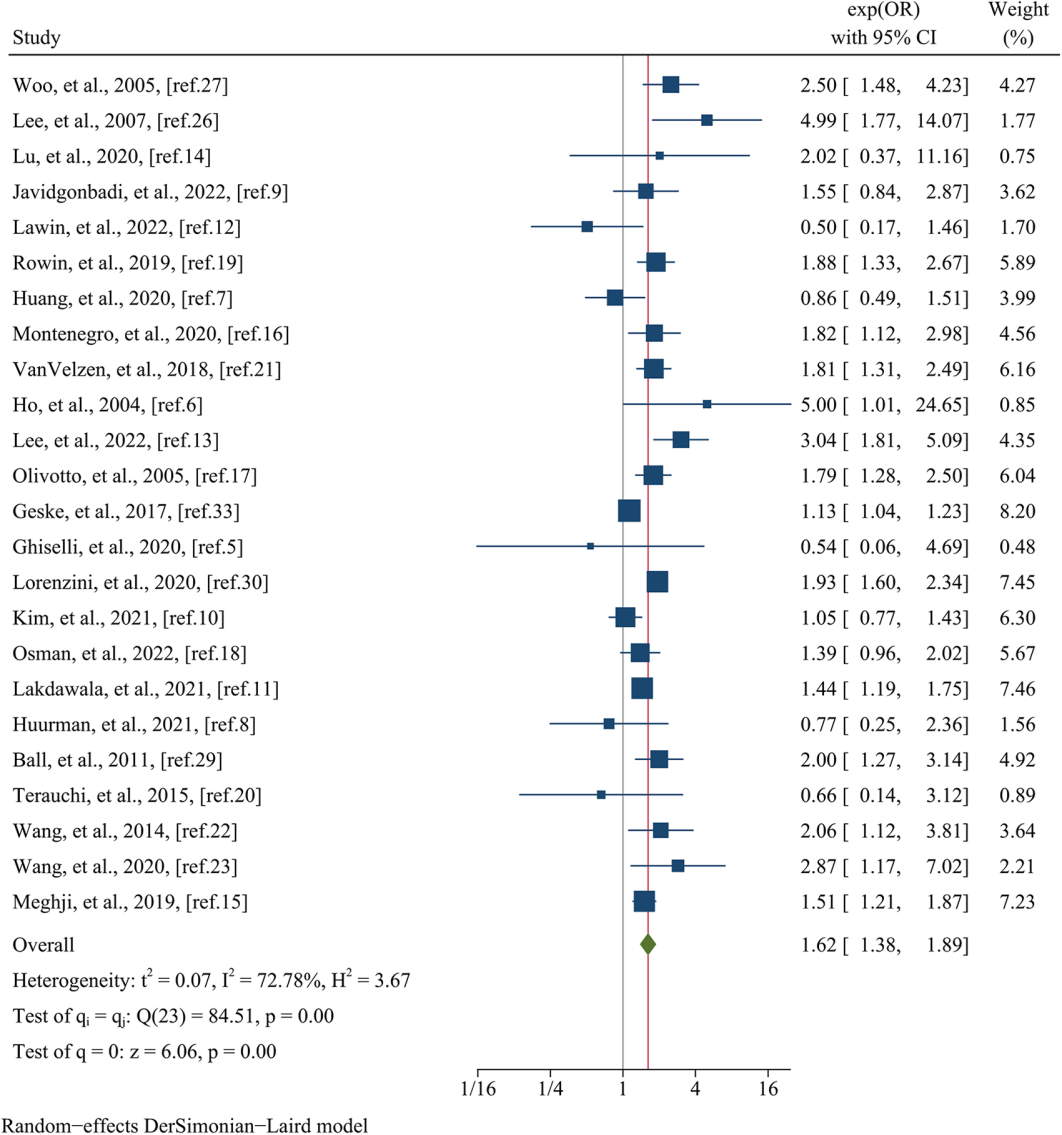
Forest plots of all–cause mortality.

**Figure 3:**
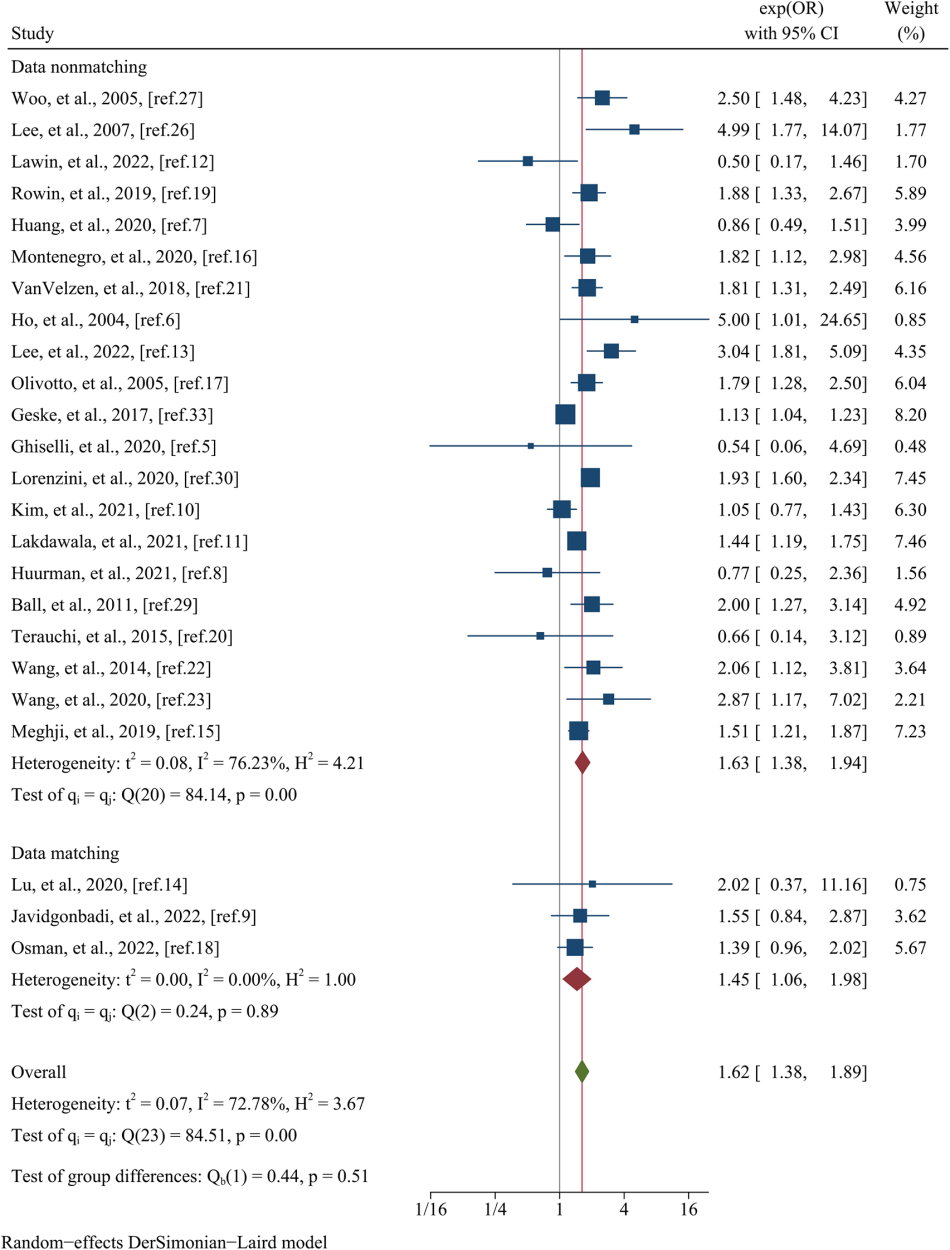
Subgroup analysis of all–cause mortality based on whether the study utilized matching or not.

Table 4 presents a comprehensive overview of the clinical outcomes observed in each study. The primary focus of the analysis was directed towards assessing all-cause mortality, which was evaluated in a total of 24 studies encompassing a substantial cohort of 36,742 patients. Visual representation of these findings can be found in Figure 2. The results revealed a significant association between female sex and an elevated risk of all-cause mortality, as evidenced by a pooled OR of 1.62 (95% CI: 1.38–1.89, *p* = 0.000, *I^2^*^ ^= 72.78%). The findings of meta-regression analysis did not yield any statistically significant differences when considering variables such as mean age (meta-regression coefficient: −0.069, *p* = 0.705), follow-up time (meta-regression coefficient: 0.472, *p* = 0.308), sample size (meta-regression coefficient: −0.267, *p* = 0.242), postoperative patients (meta-regression coefficient: 0.092, *p* = 0.672), data matching (meta-regression coefficient: 0.169, *p* = 0.657), and the type of estimated effect size in the respective studies (meta-regression coefficient: 0.085, *p* = 0.707). Although the aforementioned covariates did not exhibit statistical significance in the meta-regression analysis, we still conducted a subgroup analysis based on whether the study utilized matching or not to minimize the impact of baseline data differences. This subgroup analysis indicated no statistically significant difference (*p* = 0.51) between the matched group, consisting of three studies with a pooled OR of 1.45 (95% CI: 1.06–1.98, *I^2^* = 0.00%), and the unmatched group, comprised of 21 studies with a pooled OR of 1.38 (95% CI: 1.63–1.94, *I^2^* = 76.23%) (Figure 3).

**Table 4:** Clinical outcomes per study included in the meta-analysis.

First author	All-cause mortality		Arrhythmic endpoint		Composite endpoint	
	Female, *n*	Male, *n*	Female, *n*	Male, *n*	Female, *n*	Male, *n*
Ghiselli et al. (5)	1	5	2	1	8	10
Ho et al. (6)	8	2	–	–	–	–
Huang et al. (7)	23	32	–	–	69	66
Huurman et al. (8)	5	10	0	3	5	13
Javidgonbadi et al. (9)	50	41	7	4	–	–
Kim et al. (10)	55	178	–	–	218	495
Lakdawala et al. (11)	213	249	111	202	–	–
Lawin et al. (12)	5	11	18	15	–	–
Lee et al. (13)	32	32	8	14	42	44
Lu et al. (14)	4	2	5	9	26	18
Meghji et al. (15)	210	182	26	35	–	–
Montenegro et al. (16)	38	31	36	30	–	–
Olivotto et al. (17)	88	80	26	33	–	–
Osman et al. (18)	71	50	–	–	–	–
Rowin et al. (19)	70	65	26	61	–	–
Terauchi et al. (20)	3	5	8	7	11	7
VanVelzen et al. (21)	92	91	20	37	119	128
Wang et al. (22)	19	28	8	16	–	–
Wang et al. (23)	19	7	32	17	–	–
Lorenzini et al. (30)	230	239	46	122	314	407
First author	HR/OR	Pooled estimate and 95% CI	HR/OR	Pooled estimate and 95% CI	HR/OR	Pooled estimate and 95% CI
Geske et al. (33)	HR	1.13 (1.03–1.22)	–	–	–	–
Hutt et al. (24)	–	–	–	–	HR (male vs. female)	1.03 (0.80–1.32)
Jang et al. (25)	–	–	–	–	HR	3.31 (1.17–9.35)
Lee et al. (26)	OR	4.99 (1.77–14.08)	–	–	–	–
Woo et al. (27)	HR	2.5 (1.5–4.3)	–	–	–	–
Ho et al. (28)	–	–	HR	0.69 (0.51–0.94)	–	–
Ball et al. (29)	HR	2.0 (1.3–3.2)	–	–	–	–
Kubo et al. (31)	–	–	–	–	HR (male vs. female)	0.93 (0.54–1.06)
Choi et al. (32)	–	–	HR	2.97 (1.12–7.93)	–	–

OR, odds ratio; HR, hazard ratio; CI, confidence interval.

**Figure 4:**
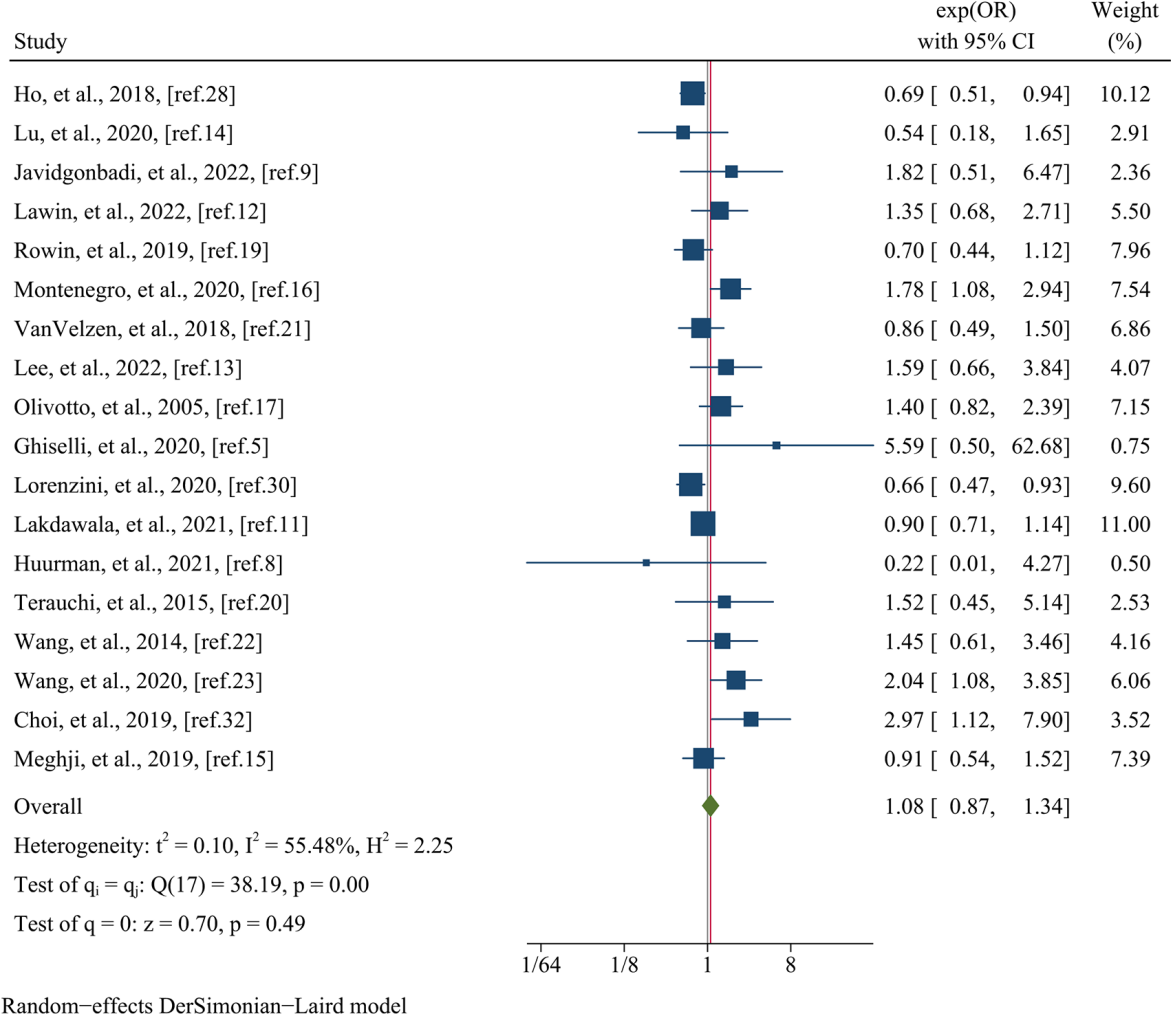
Forest plots of the arrhythmic event.

**Figure 5:**
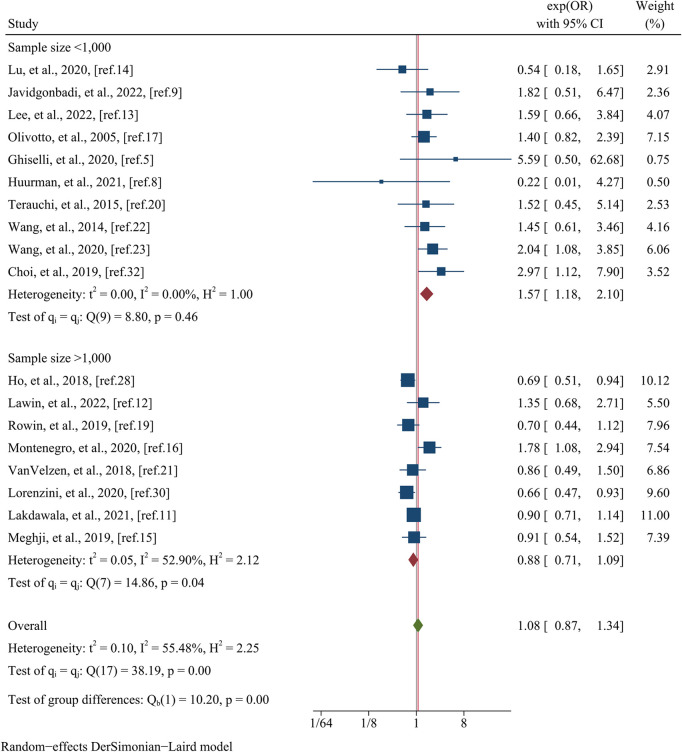
Subgroup analysis of the arrhythmic event based on sample size.

The analysis of arrhythmic endpoints encompassed data from a total of 18 studies, involving 27,947 participants (Figure 4). The investigation revealed that there was no significant difference in the occurrence of arrhythmic events between the female and male groups, as indicated by a pooled OR of 1.08 (95% CI: 0.87–1.34, *p* = 0.490, *I^2^* = 55.48%) (Figure 4). In meta-regression analysis, no significant differences were observed on the basis of the mean age (meta-regression coefficient: −0.313, *p* = 0.160), follow-up time (meta-regression coefficient: −0.042, *p* = 0.923), postoperative patients (meta-regression coefficient: −0.033, *p* = 0.914), data matching (meta-regression coefficient: −0.805, *p* = 0.168), or the type of estimated effect size of the study (meta-regression coefficient: 0.003, *p* = 0.991). However, the analysis did reveal a significant influence of sample size (meta-regression coefficient: −0.674, p = 0.002), indicating that the OR for arrhythmic endpoints between female and male groups decreased as the sample size increased. This finding provides some insight into the observed heterogeneity (*I^2^* residual = 35.57%, *p*-value for heterogeneity = 0.11). Further subgroup analysis based on sample size yielded significant results (*p* = 0.00) (Figure 5). The subgroup consisting of studies with a sample size less than 1,000 included 10 studies, with a pooled OR of 1.18 (95% CI: 1.57–2.10, *I^2^* = 0.00%). Conversely, the subgroup comprising studies with a sample size greater than 1,000 included 8 studies, with a pooled OR of 0.88 (95% CI: 0.71–1.09, *I^2^* = 52.90%).

**Figure 6:**
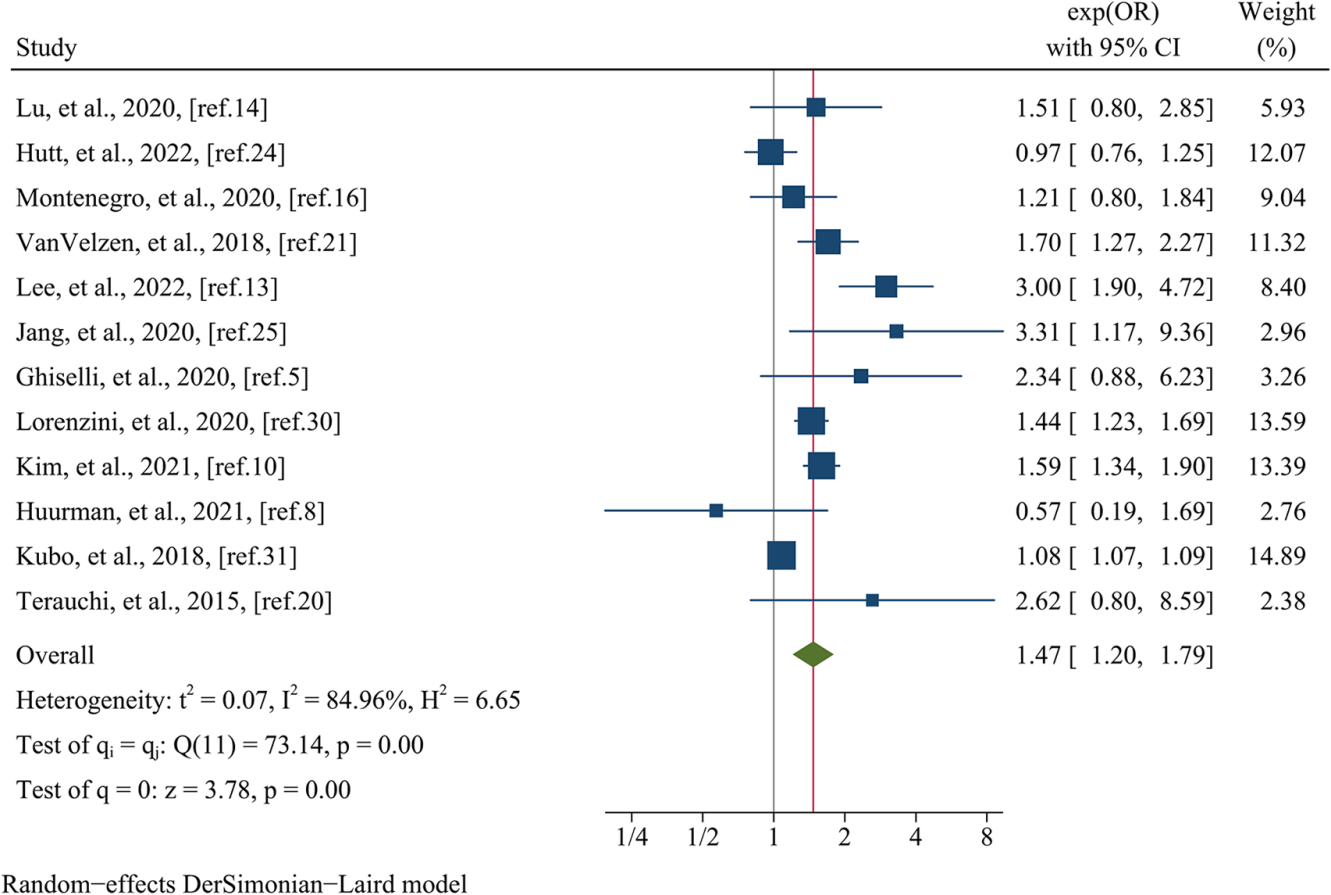
Forest plots of the composite endpoint.

**Figure 7:**
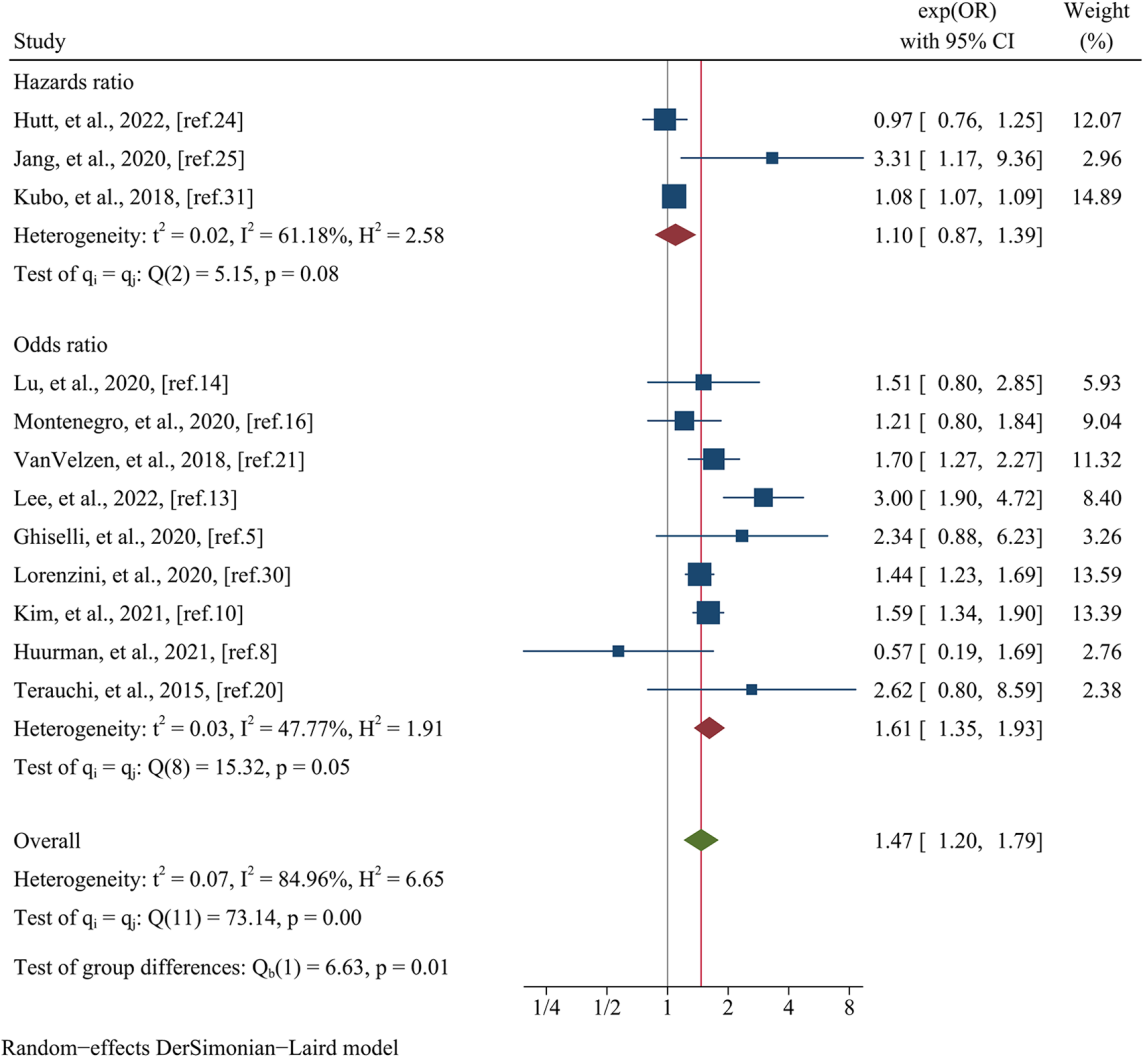
Subgroup analysis of the composite endpoint based on the type of estimated effect size.

**Figure 8:**
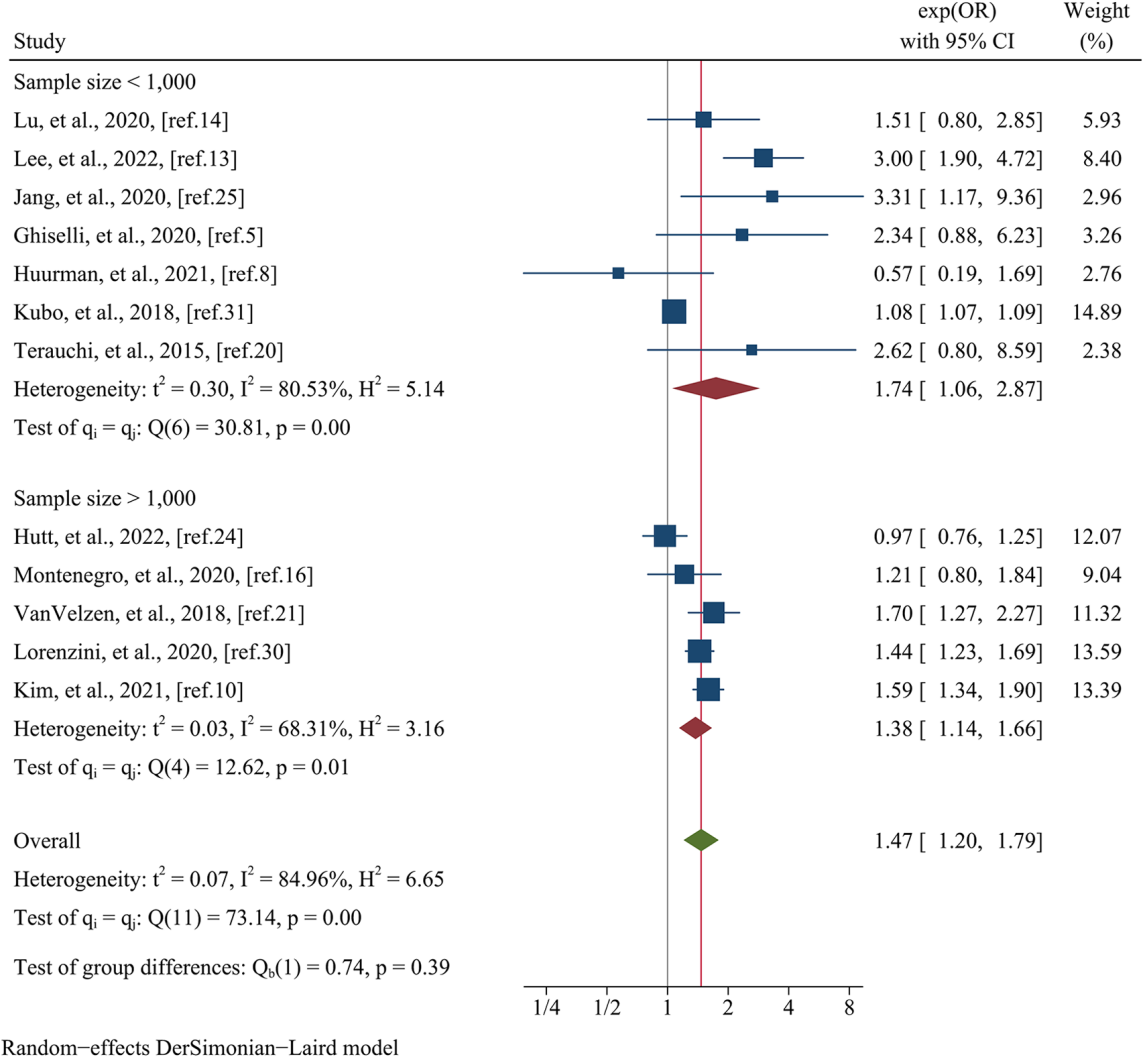
Subgroup analysis of the composite endpoint based on the sample size.

Data pertaining to the composite endpoint were extracted from a comprehensive analysis of 12 studies, encompassing a total of 16,863 patients (Figure 6). The findings from this analysis revealed a significant association between female sex and an elevated risk of experiencing the composite endpoint. This association was substantiated by a pooled OR of 1.47 (95% CI: 1.20–1.79, *p* = 0.000, *I^2^* = 84.96%) as illustrated in Figure 6. Meta-regression disclosed that variables such as data matching (meta-regression coefficient: −1.449, *p* = 0.026), postoperative patients (meta-regression coefficient: −1.351, *p* = 0.020), the type of estimated effect size (meta-regression coefficient: 0.665, *p* = 0.000), and sample size (meta-regression coefficient: −0.306, *p* = 0.027) accounted for a considerable portion of the observed heterogeneity (*I^2^* residual = 20.20%, *p* for heterogeneity = 0.281). However, it is noteworthy that covariates such as mean age (meta-regression coefficient: 0.097, *p* = 0.500) and follow-up time (meta-regression coefficient: −1.072, *p* = 0.500) did not attain statistical significance in elucidating the heterogeneity observed. Given the limited number of studies available within postoperative patients and data matching categories (only one study each), subgroup analyses based on these specific covariates were not conducted. Nevertheless, when stratified by the type of estimated effect size, the subgroup analysis revealed a statistically significant distinction between the groups (*p* = 0.01). Specifically, the group incorporating OR comprised nine studies, yielding a pooled OR of 1.61 (95% CI: 1.35–1.93, *I^2^* = 47.77%). Conversely, the group incorporating HR consisted of three studies, generating a pooled OR of 1.10 (95% CI: 0.87–1.39, *I^2^* = 61.18%) (Figure 7). Regarding subgroup analysis based on sample size, no statistically significant variation was observed between the groups (*p* = 0.39). The group encompassing studies with a sample size below 1,000 (comprising seven studies) demonstrated a pooled OR of 1.74 (95% CI: 1.06–2.87, *I^2^* = 80.53%). Conversely, the group incorporating studies with a sample size exceeding 1,000 (comprising five studies) displayed a pooled OR of 1.38 (95% CI: 1.14–1.66, *I^2^* = 68.31%) (Figure 8).

### 3.4. Sensitivity analysis

This sensitivity analysis involved systematically excluding one study at a time for each clinical outcome, allowing us to evaluate the influence of individual studies on the overall findings. Notably, none of the results exhibited significant changes upon the exclusion of any specific study (Supplementary Figure S1). These findings provide strong evidence for the stability and reliability of our results, indicating that they are not unduly influenced by the inclusion or exclusion of any particular study.

### 3.5. Publication bias

Publication bias was assessed using Egger's test, which yielded non-significant results for all-cause mortality (*p* = 0.731), arrhythmic endpoint (*p* = 0.055), and composite endpoint (*p* = 0.120). Furthermore, the funnel plot presented in Supplementary Figure S2 visually displays the distribution of studies, providing additional support for the absence of publication bias.

## 4. Discussion

This meta-analysis aimed to systematically compare and analyze the clinical outcomes between female and male patients diagnosed with HCM. The primary observations of this study indicate that female individuals with HCM exhibit a heightened risk of adverse events, specifically all-cause mortality and composite endpoint outcomes, when compared to their male counterparts. However, intriguingly, no statistically significant disparity was detected in the occurrence of arrhythmic endpoints between the two sex groups.

Two recent meta-analyses have investigated sex disparities in HCM. Angkawipa Trongtorsak et al. conducted a comprehensive meta-analysis involving 11 studies and 9,427 HCM patients (34). Their analysis focused on all-cause mortality, HCM-related mortality, and worsening heart failure or hospitalization due to heart failure as primary outcomes of interest. In our meta-analysis, we built upon their work by incorporating an additional 18 studies and a larger cohort comprising 35,250 HCM patients. Notably, our analysis included the largest single study to date, with 5,873 participants, enhancing the statistical power and precision of our estimates. Moreover, we introduced an additional endpoint related to arrhythmia, encompassing SCD, aborted SCD, appropriate shocks from ICD, and SVT/SVF. SCD remains a significant public health concern, accounting for a considerable proportion of cardiovascular deaths in developed countries (35). While the underlying causes of SCD are generally thought to be similar between the sexes, emerging evidence suggests potential sex-specific differences (35). Furthermore, there is evidence of underutilization of ICD devices among women during HCM-related hospitalizations (36). Consequently, our inclusion of the arrhythmia endpoint aims to explore potential sex disparities in this specific domain. Another meta-analysis conducted by Zhao et al. encompassed 27 cohorts and 42,365 HCM patients (37). They included outcomes of AF (5 articles with 7,453 individuals), ventricular arrhythmia (3 studies involving 7,222 patients), cardiovascular events (2–9 articles and 456–20,283 patients), deaths (5–14 articles and 9,565–31,764 patients), and composite endpoint (6 articles with 10,190 individuals). Although our study covered fewer outcomes, each outcome included more than ten articles involving substantial patient populations (16,863–36,742). Our study incorporated a comparable number of articles (29) and participants (44,677) to theirs, differences in our inclusion criteria arose due to variations in outcome measures. Furthermore, our analysis included five recently published articles from 2022, contributing an additional 7,518 patients to our comprehensive investigation (9, 12, 13, 18, 24).

HCM has consistently exhibited a male predominance, with men comprising approximately 60% of individuals in most published HCM cohorts (38). In cases of mild left ventricular hypertrophy, women may be underrepresented due to diagnostic bias, as they typically exhibit less hypertrophy (no body surface area correction) and fewer electrocardiographic abnormalities (39). MYBPC3 mutation had been reported to be associated with delayed expression of hypertrophy (20). Unfortunately, this study did not find any difference between the two groups regarding MYBPC3 due to the limitation of sample size. In animal models, estrogen can inhibit cardiac hypertrophy through epigenetic modifications, which may account for a delay in disease onset (40).Women tend to be diagnosed at an older age compared to men, with a mean age at diagnosis of 61.4 ± 15.0 years vs. 51.8 ± 13.6 years, respectively (41). Thus, the inclusion of predominantly older female participants in these included studies reflects this demographic characteristic. Our analysis also revealed that the MWT was lower in the female group compared to the male group. As men have a higher absolute body surface area (BSA) and weight, their BSA- and weight-indexed MWT were similar to those of women (42). It is worth noting that the limited availability of indexed MWT values in the included studies restricted our ability to comprehensively evaluate this parameter. Furthermore, a higher proportion of female patients presented with a LVOTG greater than or equal to 30 mmHg compared to the male group. This observation can be attributed to the characteristic anatomical and physiological differences between the sexes, as women with HCM tend to exhibit a smaller left ventricular cavity size and higher left ventricular contractility (43). Patients with HOCM are characterized by ventricular hypertrophy, myocardial fibrosis, and impaired diastolic function, which often contribute to an increased risk of SCD (44). The compensatory mechanism of left atrial (LA) enlargement to modulate left ventricular filling pressure is impaired in female patients with HOCM due to their heightened susceptibility to myocardial fibrosis (45). Moreover, Chen YZ et al. reported that males with HCM demonstrated favorable reverse remodeling with greater regression of left ventricular mass after alcohol septal ablation compared to female patients (46). The presence of myocardial remodeling and fibrosis in HCM has been associated with impaired diastolic function, which may contribute to a higher predisposition to heart failure in women compared to men (44). Consequently, it is not surprising that the included studies reported a higher proportion of females with NYHA class III/IV, indicating more advanced stages of HF, as compared to males. The observed baseline differences among studies can be attributed to variations in inclusion criteria. Nevertheless, it is important to recognize that inherent sex-related differences also contribute to the disparities in demographic and clinical characteristics in HCM.

This study revealed a significant association between female sex and an elevated risk of all-cause mortality compared to male sex in patients with HCM. These findings are consistent with previous meta-analyses that have reported similar outcomes (34, 37). Subgroup analysis based on data matching demonstrated that even within the data matching group where heterogeneity was absent, women continued to exhibit a higher risk of all-cause mortality compared to men. For instance, the comprehensive study conducted by Neal K. Lakdawala et al. encompassing a substantial population and a median follow-up time of 7.7 years, demonstrated a higher all-cause mortality rate in women compared to men (9.6% vs. 6.8%) (11). While Dai-Yin Lu et al. observed that women and men had comparable mortality rates (2% vs. 1%), women displayed a higher incidence of HF in comparison to men (8% vs. 4%) (14). Davood Javidgonbadi et al. revealed that females had a greater disease-related mortality rate than males (2.9% vs. 1.5%), with a prevalence of excess female deaths occurring in cases of HF and acute myocardial infarctions (9). Conversely, non-disease-related deaths were more frequently observed in males (6 patients vs. 18 patients). Another study by Mohammed Osman et al. utilizing propensity score matching, found no disparity in mortality between women and men (3% vs. 2.4%), albeit with a limited 30-day follow-up (18). In the context of HCM, women tend to receive their diagnosis at an older age, partly attributed to genetic factors (11). Furthermore, fluctuations in hormonal levels during the perimenopausal phase may exacerbate symptoms in female patients with apical HCM, potentially offering some relief during the postmenopausal period (47). This factor may also influence the timing of medical visits for female patients. Among the known causal genes, MYH7 and MYBPC3 are the two most common, collectively responsible for approximately half of the patients with familial HCM (48). Patients with MYH7 mutations were more likely to progress to end stage HF compare to those with MYBPC3 variants (40). In summary, female patients with HCM experience delayed diagnosis, a heightened incidence of heart failure, and an increased disease-related mortality rate, consequently resulting in an elevated all-cause mortality rate compared to their male counterparts.

The analysis of the arrhythmic endpoint did not yield any statistically significant differences between the female and male groups. This finding aligns with previous meta-analyses that have reported a lack of association between female sex and an increased risk of ventricular arrhythmias and SCD (37). Subgroup analysis based on sample size revealed a lack of heterogeneity within the subgroup comprising studies with a sample size of less than 1,000, yet women exhibited a higher risk of the arrhythmic endpoint compared to men. The sample size range included in this subgroup analysis ranged from 50 to 969. Among the studies included, the most notable investigation was conducted by Iacopo Olivotto et al., encompassing 969 patients with a median follow-up time of 6.2 years (17). Their findings demonstrated that the incidence of SCD was similar between men and women (7% vs. 6%). However, another study suggested a higher incidence rate ratio of SCD in males compared to females, particularly among patients under the age of 35 (35). Additionally, Sri Harsha Patlolla et al. identified underutilization of ICD devices in women during HCM hospitalizations (36). However, we found no statistically significant difference in the use of ICD between the two groups during the course of HCM. Our subgroup analysis, which primarily focused on studies with smaller sample sizes, supports the notion that there is no significant difference in the arrhythmic endpoint between female and male patients with HCM.

The female group exhibited a significant association with an elevated risk of the composite endpoint. In contrast, a prior study reported no significant difference in the composite endpoint between the female and male groups (37). Their composite endpoint, which comprised six studies, encompassed HF hospitalization or HCM-related events, SCD, and death. Our subgroup analysis, stratified by the type of estimated effect size, revealed lower heterogeneity in the OR group, with women still demonstrating a higher risk of all-cause mortality compared to men. As previously noted, females exhibited a higher incidence of HF hospitalization and a greater all-cause mortality rate than males. Conversely, there was no notable distinction in the arrhythmic endpoint between the two groups. Consequently, we deduced that the increased risk of the composite endpoint in women was reasonable and supported by the observed findings.

In summary, we believe that women have worse clinical outcomes. Compared to male patients, females are diagnosed later, and the high mutation rate of MYH7, which leads to more severe HF, may be the cause. Further research is needed to determine whether the high mutation rate of MYBPC3 and the low utilization rate of ICD affect the prognosis of female patients.

## 5. Limitations

This meta-analysis is subject to several significant limitations that warrant careful consideration. Firstly, it is important to note that all the studies included in our analysis were of observational design, thereby rendering them susceptible to inherent selection bias and potential confounding factors. Secondly, the adoption of data matching techniques was limited among the included studies, leading to substantial heterogeneity in baseline characteristics across the dataset. Consequently, the interpretation of our findings should be approached with caution, considering the potential impact of these baseline differences. Thirdly, although rigorous measures were taken, involving independent data extraction by two researchers and subsequent discussion among a panel of three experts, the inherent variability in outcome reporting among studies may introduce errors in data extraction. Thus, the possibility of data extraction inaccuracies should be acknowledged. Lastly, it is worth noting that the observed heterogeneity in our results, while partially explored through regression analysis, may still be influenced by unidentified factors. Hence, the strength and generalizability of our findings may be influenced by the presence of unaccounted sources of heterogeneity.

## 6. Conclusions and implications

The association between female sex and poorer clinical outcomes in hypertrophic cardiomyopathy (HCM) appears to be of significance. However, in order to establish robust and reliable conclusions, it is imperative to underscore the necessity for further investigation involving paired populations, given the observed differences in baseline data across the included studies. Moreover, it is crucial to uphold the principle that “Everyone is equal to the wise one,” irrespective of sex (49). Traditionally, heart disease has been predominantly perceived as a condition affecting men, highlighting longstanding disparities in healthcare that persist to this day and have been further underscored by the COVID-19 pandemic (50). Consequently, it is imperative to critically reevaluate disease definitions and enhance awareness to mitigate delays in the diagnosis and treatment of HCM in women, thereby fostering equitable healthcare practices (41).

## Data Availability

The original contributions presented in the study are included in the article/**Supplementary Material**, further inquiries can be directed to the corresponding author.
